# Correlation between Psychosocial Distress and Quality of Life in Patients with Nasopharyngeal Carcinoma following Radiotherapy

**DOI:** 10.1155/2018/3625302

**Published:** 2018-08-06

**Authors:** Xiaolan Wang, Yue Lv, Wen Li, Chen Gan, Haijun Chen, Yingying Liu, Herta H. Chao, Chiang-Shan R. Li, Huaidong Cheng

**Affiliations:** ^1^Department of Oncology, The Second Affiliated Hospital of Anhui Medical University, No. 678 Furong Road, Hefei, Anhui 230601, China; ^2^Cancer Hospital, Hefei Institutes of Physical Science, Chinese Academy of Sciences, No. 350, Shushan Hu Road, Hefei, Anhui 230031, China; ^3^Cancer Center, VA Connecticut Healthcare System, West Haven, CT 06516, USA; ^4^Department of Psychiatry, Yale University School of Medicine, New Haven, CT 06520, USA

## Abstract

The aim of this study was to investigate the relationship between psychosocial distress and quality of life (QOL) in patients with nasopharyngeal carcinoma (NPC) after radiotherapy. Fifty-three patients with an initial diagnosis of NPC were enrolled in this study. The psychological Distress Thermometer (DT) and Functional Assessment of Cancer Therapy-Head & Neck (FACT-H&N) were conducted before and after radiotherapy in NPC patients. We compared the differences in psychological distress and QOL before and after radiotherapy and analyzed the correlation between psychological distress and QOL after radiotherapy. The performance on the DT was 6.60 ± 1.42 and 2.81 ± 1.43 before and after chemotherapy, respectively, with a significant difference between the time points (t = -13.73,* P *< 0.01). The performance on the FACT-H&N was 68.30 ± 6.14 and 39.84 ± 6.14 before and after chemotherapy, respectively, with a significant difference between the time points (t = -19.9,* P*< 0.01). There was a significant negative correlation between the DT score and the FACT-H&N score (r = -3.64,* P*< 0.01). Patients with NPC experience different degrees of psychological distress, an important factor that affects quality of life, after radiotherapy.

## 1. Introduction

Nasopharyngeal carcinoma (NPC) is one of the most common malignancies in the southern region of China. One study that assessed the rates of invasive malignant tumor diagnosed in 2015 found that the incidence rates of NPC were substantially higher in males than in females [1]. Radiation therapy is one of the most important treatments for NPC and can effectively extend the survival of NPC patients [2]. However, NPC patients may emerge from therapy with a range of side effects, including reactions of the mucous membranes, pain, fatigue, dysphasia, and difficulty swallowing. These side effects may result in certain emotional responses, thereby influencing the patient's quality of life (QOL) [3]. Psychological distress is caused by multifaceted adverse emotional experiences that have psychological (cognitive, behavioral, and emotional) and social side effects. In addition, the nature of the spirit may affect an individual's ability to respond effectively to cancer, physical symptoms, and treatment side effects [4]. Psychological distress is widespread in many cancer patients [5], and previous studies have suggested that the diagnosis and treatment of NPC can lead to the occurrence of psychological distress [6].

The QOL and prognosis of cancer patients are significantly affected by psychological distress. Cancer patients often experience psychological problems such as severe depression and anxiety [7]. Physical, social, cognitive, psychological, and emotional issues, as well as physical symptoms such as pain, nausea, vomiting, and fatigue, all affect QOL [8]. Several studies have found that patients with NPC have psychological distress after radiotherapy, with approximately 13% of patients suffering from severe psychological distress [9]. Buchmann et al. [10] found that patients with head and neck cancer, including NPC, had a higher incidence of psychological distress, including a self-reported history of depression, concerned relatives and friends, and emotional and physical problems. NPC patients often experience head and neck pain, skin and mucous membrane reactions, language issues and dysphagia, hearing loss, social difficulties, dry mouth, cough, phlegm, sensory discomfort, and other problems that decrease QOL during radiation therapy [11, 12]. However, there have been no studies focusing on the correlation between psychological distress and QOL in patients with NPC after radiotherapy.

In the present study, 53 patients with newly diagnosed NPC were assessed. The psychological Distress Thermometer (DT) and Functional Assessment of Cancer Therapy-Head & Neck (FACT-H&N) were performed before and after radiotherapy. We attempted to understand the incidence of psychological distress in patients with NPC after radiotherapy and its correlation with QOL.

## 2. Materials and Methods

### 2.1. Clinical Data of Study Participants

A total of 53 patients pathologically diagnosed with NPC were enrolled in the study at the Department of Oncology at the Second Affiliated Hospital of Anhui Medical University between January 2016 and March 2017. Among them, 40 participants were male and 13 were female. The participants ranged in age from 12 to 79 years, with a mean age of 7.77 ± 3.92 years and a median age of 50 years. The participants had a mean of 7.77 ± 3.92 years of education. Seven cases were stage II, 32 cases were stage III, and 14 cases were stage IIIb NPC. The mean Karnofsky Performance Status (KPS) score was 86.98 ± 7.23.

The inclusion criteria were as follows: (1) pathological diagnosis of NPC; (2) age ≥ 18 years; and (3) evidence of normal cognition and ability to communicate using normal language and words.

The exclusion criteria were as follows: (1) patients being too weak to fill out questionnaires; (2) anxiety, depression, delusions, or other psychiatric symptoms; (3) use of medication to improve cognitive function; (4) clinically diagnosed dementia; or (5) other physical or psychological disorders that may lead to cognitive dysfunction. The Research Ethics Committee of the Second Affiliated Hospital of Anhui Medical University approved the study (ethical code: 20131028), and all participants provided informed consent prior to the beginning of the study. The DT and FACT-H&N questionnaires were approved by the ethics board and complied with ethical standards.

### 2.2. Methods

Data on age, education, KPS, and tumor stage of the patients were gathered and statistically analyzed. The DT and FACT-H&N surveys were all administered within one week before and after radiation therapy.

### 2.3. The Evaluation of Psychological Distress

The DT[13] were used to assess psychological distress in patients with NPC before and after radiotherapy. DT includes 11 scales that range from 0 to 10 (0: no pain, 10: extreme pain), which covers five major aspects: practical, family, emotional, physical problems, and religious beliefs. For example: if one patient had no problem with all these five aspects (practical, family, emotional, physical problems, and religious beliefs), the score of DT is 0.

### 2.4. The Evaluation of QOL

In this study, the FACT-H&N scale was used to evaluate the QOL of patients with NPC before and after radiotherapy. FACT-H&N was designed to assess the QOL of patients with head and neck tumors[14]. The scale includes two parts for head and neck tumors: the FACT-G, a general malignant tumor module, and a specific module. The scale is divided into five fields, including physical health status, social/family health status, emotional condition, functional status, and head and neck attachment, with 39 questions in total. All questions are scored on scales ranging from 0 to 4 (0: ‘almost never', 4: ‘very often'). Higher scores on the FACT-H&N are indicative of a better quality of life. Chinese researchers have previously been successful at using the FACT-H&N to measure the QOL in patients with NPC [15].

### 2.5. Statistical Analysis

SPSS software (version 22.0, Chicago, IL) was used for statistical analysis. Student's* t*-tests were used to analyze continuous variables such as age and education. Comparisons between groups were assessed using independent samples* t*-tests. Linear correlation analysis was performed using a Pearson correlation analysis. For all statistically matched two-tailed probability tests, statistical significance was defined at a level of* P*< 0.05.

## 3. Results

### 3.1. Comparison of DT and FACT-H&N Scores

As shown in Table 1, the DT scores of NPC patients were 6.60 ± 1.42 and 2.81 ± 1.43 before and after radiotherapy, respectively, with a significant difference between the time points (t = -13.73,* P*< 0.01). The FACT-H&N scores of NPC patients were 68.30 ± 6.14 and 39.84 ± 6.14 before and after chemotherapy, respectively, with a significant difference between the time points (t = -19.9,* P*< 0.01).

### 3.2. Correlation Analysis of Psychological Distress and QOL in Patients with Nasopharyngeal Carcinoma

**Figure 1:**
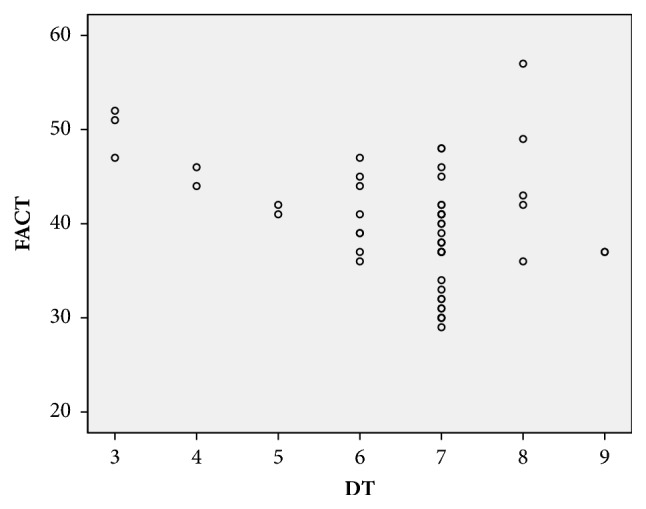
Correlation analysis of DT and FACT in nasopharyngeal carcinoma patients.

As shown in Figure 1, there was a significant negative correlation between the DT and FACT-H&N scores (r = -3.64,* P* = 0.007 < 0.01) in nasopharyngeal carcinoma patients after radiotherapy. These results indicate that the higher the DT score, the worse the QOL for the patient.

## 4. Discussion

Psychological distress in cancer patients mainly manifests as depression, anxiety, and adaptive disability and can be associated with both cancer and its treatment. A longitudinal prospective study found that psychological distress among cancer patients was associated with QOL, such that patients with moderate to severe psychological distress had a lower QOL [16]. Radiotherapy is one of the most important treatments for patients with NPC. It is thought that serious psychological and physiological stress are caused by long radiotherapy periods, high doses of radiotherapy, and therapy side effects [17]. Compared to that before radiotherapy, our study found that the psychological distress of patients with NPC increased significantly after radiotherapy, that QOL decreased significantly after radiotherapy, and that there was a correlation between the two time points.

The duration of radiotherapy treatment for patients with NPC can be very long, and patients may lack awareness regarding the therapy. After radiotherapy, patients may experience varying degrees of side effects. In patients with NPC, the economic burden of treatment coupled with the fear of cancer and the prognosis following treatment frequently leads to psychological distress [10]. NPC patients may find it difficult to accept their sickness or may be unable to communicate effectively with family members to obtain psychological support. Also, the physical and economic problems caused by the body and by treatment can lead to depression, which is closely related to psychological distress [18]. Psychological distress seriously affects the QOL of patients with NPC, which can negatively affect their treatment and prognosis [19]. In a study of 81 cases of head and neck tumors, Ninu et al. [20] found that 41% of patients showed severe psychological distress (DT > 5), which in turn affected their QOL.

As the technology for radiotherapy for the treatment of NPC improves, the survival duration of NPC patients increases. As a result, the QOL of NPC patients has become a greater concern. There are many factors that influence the QOL of patients with NPC, such as the disease itself [21], age and gender [22], socioeconomic factors [23], and psychological factors [24]. However, the correlation between psychological distress and QOL in NPC patients had not been studied.

This is the first study to use the DT and FACT-H&N scales to study the relationship between psychological distress and QOL before and after radiotherapy therapy in NPC patients. We found psychological distress to be negatively correlated with QOL in NPC patients after radiotherapy; i.e., the more severe the psychological distress, the lower the QOL. Therefore, reducing psychological distress may be an important means to improving the QOL of NPC survivors. Nasopharyngeal carcinoma patients should be provided with assistance following radiotherapy to maintain their quality of life.

This study has several limitations. First, this was a small study with a cross-sectional design. A larger sample size and a longitudinal study design are required for future studies. Second, this study only included individuals from the Han population; therefore, it is unclear whether the results can be applied to other populations. Third, the lack of a healthy control group in this study means that the results cannot be completely ascertained.

In conclusion, this study found that patients with NPC have varying degrees of psychological distress after radiotherapy and that the degree of psychological distress was negatively correlated with QOL. These results suggest that psychological distress may be an important factor affecting the QOL in NPC patients after radiotherapy. This study provides a theoretical basis for improving the QOL in patients with nasopharyngeal carcinoma.

## Figures and Tables

**Table 1:** Comparison of DT and FACT-H&N scores in patients with nasopharyngeal carcinoma before and after radiotherapy.

Group	DT	FACT-H&N
Before radiotherapy	2.81 ± 1.43	68.30 ± 6.14
After radiotherapy	6.60 ± 1.42*∗*	39.84 ± 6.14*∗*

Note: *∗*:* P *< 0.01; DT, psychological distress thermometer; FACT-H&N, Functional Assessment of Cancer Therapy-Head & Neck.

## Data Availability

The data used to support the findings of this study are available from the corresponding author upon request.
